# First report of *Ophiotaenia* sp. in frogs (*Amietophrynus kassasii*) from Egypt and *in vitro* anticestodal activity of *Sinularia* sp. extract

**DOI:** 10.14202/vetworld.2025.3815-3825

**Published:** 2025-12-13

**Authors:** Barakat Shehata Abd elmaleck, Mahmoud Abdelhamid, Abdallah Alian, Hind Alzaylaee, George D. Zouganelis, Gaber El-Saber Batiha, Marwa Adel Thabet, Fatma A. S. Anwar

**Affiliations:** 1 Department of Zoology and Entomology, Faculty of Science, New Valley University, El Kharga, New Valley, Egypt; 2 Department of Parasitology, Faculty of Veterinary Medicine, Aswan University, Aswan, 81528, Egypt; 3 Department of Zoology, Faculty of Science, Al-Azhar University, Assiut, Egypt; 4 Department of Biology, College of Science, Princess Nourah bint Abdulrahman University, P.O. Box 84428, Riyadh, 11671, Saudi Arabia; 5 Department of Biomedical and Forensic Sciences, School of Sciences, College of Science & Engineering, University of Derby, Kedleston Rd, DE22 1GB, Derby, United Kingdom; 6 Department of Pharmacology and Therapeutics, Faculty of Veterinary Medicine, Damanhour University, Damanhour, 2251, AlBeheira, Egypt; 7 Department Zoology and Entomology, Faculty of Science, Assiut University, Assiut, 71516, Egypt

**Keywords:** *Amietophrynus kassasii*, anthelmintic, cestode, *Ophiotaenia*, scanning electron microscopy, *Sinularia*

## Abstract

**Background and Aim:**

*Ophiotaenia* species are globally distributed proteocephalidean cestodes that commonly parasitize amphibians and reptiles. Despite the ecological importance of frogs in controlling insect populations and maintaining food-web stability, data on cestode infections in Egyptian amphibians remain scarce. This study provides the first documentation of *Ophiotaenia* sp. infecting *Amietophrynus kassasii* in Egypt and evaluating the *in vitro* anticestodal activity of *Sinularia* sp. extract against adult tapeworms.

**Materials and Methods:**

A total of 85 frogs were collected from freshwater ponds in New Valley Governorate, Egypt, between February and September 2024. Intestines were examined for cestodes, which were identified morphologically using light microscopy and scanning electron microscopy (SEM). Soft coral *Sinularia* sp. extract was prepared by methanolic extraction, and three concentrations (25, 50, 100 µg/mL) were assessed for anticestodal efficacy using motility, paralysis, and mortality endpoints. Tapeworms from the control and highest-dose groups were subjected to SEM to evaluate tegumental alterations.

**Results:**

*Ophiotaenia* sp. infection was detected in 5 of 85 frogs (5.9%), with a notably high mean intensity of 70 parasites per host. The recovered cestodes measured 12–30 mm × 0.7–0.9 mm, featuring a scolex with two spherical suckers and a distinct apical organ. Gut-content analysis of infected frogs revealed coleopteran, orthopteran, and hymenopteran insects as probable intermediate or paratenic hosts. *Sinularia* sp. extract exhibited clear dose-dependent anticestodal activity. Mortality occurred at 7.58 ± 0.15 h (25 µg/mL), 5.79 ± 0.08 h (50 µg/mL), and 4.247 ± 0.09 h (100 µg/mL), compared with 70.39 ± 1.23 h in controls. SEM analysis of treated cestodes showed profound tegumental erosion, sucker shrinkage, cirrus sac constriction, and proglottid contraction, indicating severe structural disruption.

**Conclusion:**

This study documents the first occurrence of *Ophiotaenia* sp. in *A. kassasii* in Egypt and provides evidence that *Sinularia* sp. extract possesses strong, dose-dependent anticestodal properties. The pronounced tegumental damage observed suggests potent cestocidal mechanisms. These findings offer new insights into amphibian parasitology in Egypt and support the potential development of marine-derived natural products as alternative anthelmintics.

## INTRODUCTION

The frogs *Amietophrynus kassasii* are commonly found across stagnant freshwater habitats within Egypt’s Nile Valley and Delta regions [1]. Members of the genus *Ophiotaenia*, originally described under the order Proteocephalidea Mola, 1928 and now placed within the order Onychophora (Caira, Jensen, Waeschenbach, Olson, and Littlewood, 2004), represent a diverse group of cestodes widely distributed among reptiles, small fish, and amphibians, particularly frogs, with more than 130 recognized species [2, 3]. In Africa, most records of proteocephalidean tapeworms belonging to *Ophiotaenia* date back to the early 20^th^ century. The genus *Ophiotaenia* La Rue, 1911 is the second most species-rich group within the Proteocephalidae, comprising over 60 species that parasitize reptiles worldwide [4]. Despite this diversity, the true number of proteocephalids infecting amphibians and reptiles is likely underestimated, largely due to the high biodiversity of hosts and the limited research attention historically devoted to their parasitic fauna [4–7].

*Ophiotaenia* is considered a non-monophyletic assemblage of tapeworms capable of parasitizing a wide range of amphibian and reptilian hosts across the globe. Although species are generally thought to be host-specific within tetrapod groups, they are particularly significant because they serve as valuable model taxa for studies of host–parasite coevolution, diversification, and the biogeographical processes shaping parasite distribution. A striking example is the detection of the North American species *Ophiotaenia perspicua* (La Rue, 1911), originally from *Nerodia rhombifer*, in Europe, an instance of “parasite spillover” that highlights how global trade and the movement of animals can introduce novel parasite lineages into new ecosystems [8].

While the life cycles of a few *Ophiotaenia* species have been partially elucidated, the developmental biology of many, particularly Neotropical taxa, remains unknown. Copepods serve as the first intermediate hosts, whereas insects, tadpoles, and small fish may act as paratenic or secondary intermediate hosts [9].

Marine natural products represent an important reservoir of bioactive compounds derived from marine microorganisms, plants, and animals. These compounds possess diverse pharmacological activities, including antibacterial, antifungal, anti-inflammatory, and antiviral properties [10–12]. Within this context, soft corals of the genus *Sinularia* are notable for producing a wide variety of biologically active metabolites, such as diterpenes, steroids, and fatty acids, conferring antibacterial, anti-inflammatory, and antioxidant properties [13]. These compounds play essential ecological roles as chemical defenses, mediate predator–prey interactions, and hold considerable promise as sources of novel therapeutic agents [14].

Although helminth infections in amphibians have been documented in several regions of the world, information on proteocephalidean cestodes, particularly those belonging to the genus *Ophiotaenia*, remains extremely limited across North Africa and the Middle East. Existing surveys in Egypt focus largely on amphibian biodiversity, while parasitological investigations are outdated, geographically restricted, or incomplete. No previous study has reported *Ophiotaenia* species parasitizing *A. kassasii*, leaving a significant gap in understanding the distribution, host range, and morphological variability of these cestodes in Egyptian amphibians. Furthermore, most available data on *Ophiotaenia* life cycles and intermediate hosts remain fragmented, hindering ecological interpretation of transmission dynamics in local freshwater ecosystems. In parallel, despite global interest in marine-derived natural products for antiparasitic drug discovery, the anticestodal potential of *Sinularia*, a chemically rich soft coral genus, has not been evaluated against frog-infecting cestodes. The absence of baseline epidemiological data, combined with the lack of experimental evidence on natural marine extracts against *Ophiotaenia* species, highlights the need for integrative parasitological and pharmacological research.

The present study was designed to address these critical knowledge gaps by conducting the first investigation of *Ophiotaenia* species infection in *A. kassasii* frogs from the New Valley Governorate, Egypt, supported by detailed morphological and SEM examination for accurate species-level characterization. Additionally, this work aimed to evaluate the *in vitro* anticestodal efficacy of methanolic *Sinularia*sp. extract against adult *Ophiotaenia* specimens using motility, paralysis, and mortality indicators, coupled with ultrastructural assessment of tegumental alterations induced by treatment. By integrating ecological, taxonomic, and pharmacological approaches, this study seeks to generate foundational epidemiological data, elucidate key morphological traits of the isolated cestodes, and explore the therapeutic potential of *Sinularia*-derived bioactive metabolites as alternative anthelmintic candidates. Ultimately, the findings aim to enhance understanding of cestode–amphibian interactions in Egypt and contribute to the growing field of marine natural-product-based antiparasitic research.

## MATERIALS AND METHODS

### Ethical approval

All procedures involving amphibian collection, handling, transportation, and laboratory experimentation were conducted in strict accordance with the ethical guidelines for the care and use of animals in scientific research. The study protocol was reviewed and approved by the Animal Care and Use Committee of the Faculty of Sciences, Assiut University, Egypt (Approval No. 01.2025.0004). Permission for the collection of *A. kassasii* specimens from natural freshwater habitats in the New Valley Governorate was granted by the local environmental authorities.

Frogs were collected and handled using non-invasive methods to minimize stress and avoid unnecessary injury. Only freshly dead frogs were used for parasitological examination, and no animals were euthanized solely for research purposes. All dissections were carried out by trained personnel under controlled laboratory conditions using sterile instruments to ensure animal welfare and biosafety.

Experimental procedures involving cestodes isolated from the frog intestines complied with national and international ethical standards, including the Guide for the Care and Use of Laboratory Animals (National Research Council, USA), the World Organization for Animal Health standards for animal welfare, and the animal research: Reporting of *In Vivo* experiments guidelines for transparent reporting of animal-based research. All samples were disposed of in accordance with institutional biosafety and environmental regulations.

### Study period and location

**Figure 1:**
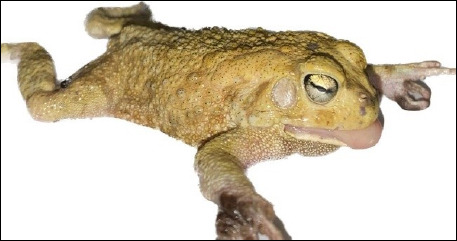
The frogs (*Amietophrynus kassasii*) appear yellowish with brown spots on their back, besides dark to black dots on the abdomen of their forelimb toes. The average length and width were 11–13.5 and 1.8–2.7 cm, respectively.

The study was conducted from February to September 2024. A total of 85 *A. kassasii* frogs (Figure 1) were collected from freshwater ponds located in the New Valley Governorate, Egypt (25.5°N, 29.5°E). These semi-permanent ponds, primarily used for irrigation and livestock watering, support dense aquatic vegetation dominated by *Typha* and *Phragmites* species, providing suitable habitats for amphibians and associated invertebrates.

Environmental parameters, including temperature (24°C–30°C), pH (7.0–7.8), and dissolved oxygen (6–8 mg/L), were recorded and found to be within normal freshwater ranges. Sampling was conducted during spring and early summer to coincide with periods of high amphibian activity and increased parasite transmission potential. Freshly dead frogs were transported to the Zoology Department laboratory, New Valley University, for parasitological examination. Intestines were dissected, placed in saline solution, and screened under a stereomicroscope. Recovered tapeworms were thoroughly washed in 0.85% saline solution for subsequent morphological identification and *in vitro* assays.

### Morphological examination of tapeworms

Whole-mount preparations of isolated cestodes were stained with acetic acid-alum carmine, followed by dehydration through ascending ethanol concentrations. Specimens were differentiated in acid alcohol (1 mL concentrated HCl per liter of 70% ethanol), dehydrated fully through 70%–100% ethanol, and cleared in xylene. Mounting was performed using Canada balsam under a coverslip, and slides were dried at 37°C before examination under light microscopy [15].

### Preparation and chemical profiling of *Sinularia* sp. extract

Soft coral samples of *Sinularia* sp. were collected from the Egyptian Red Sea coast and transported immediately to the laboratory. Approximately 250 g of tissue was cut into small pieces and extracted in methanol at room temperature. The mixture was filtered (Whatman, Merck, Germany), and the solvent was evaporated using a rotary evaporator at 50°C, yielding 4 g of crude methanolic extract.

The resulting extract underwent preliminary chemical screening following established protocols to determine its major chemical constituents [16].

### *In vitro* anticestodal assay

Adult *Ophiotaenia* sp. tapeworms (n = 6) were incubated in Petri dishes containing *Sinularia* sp. extract at concentrations of 25, 50, and 100 µg/mL (triplicate dishes per concentration). Controls were maintained in phosphate-buffered saline (PBS) without extract. All experiments were performed at 37°C ± 1°C. Tapeworm motility, paralysis, and mortality were recorded at 30-min intervals to assess cestocidal efficacy [17].

### Scanning electron microscopy (SEM)

Tapeworms from the control group and those exposed to the highest effective concentration of *Sinularia* sp. extract were prepared for SEM analysis. Samples were fixed in 5% glutaraldehyde for 24 h, rinsed 3 times with PBS, post-fixed in osmium tetroxide for 1.5 h, and dehydrated through 90%, 95%, and 100% ethanol. Specimens were mounted on metallic stubs, sputter-coated with gold–palladium and examined using a JOEL JSM-T200 SEM (JOEL, Japan) operating at 20 kV [18].

### Statistical analysis

Data were expressed as mean ± standard error. Differences among treatments were evaluated using one-way analysis of variance, followed by Duncan’s *post hoc* test using the Statistical Package for the Social Sciences version 20 (IBM Corp., NY, USA). Statistical significance was set at p < 0.05. Epidemiological descriptors, including infection rate, mean intensity, and abundance, were calculated using Quantitative Parasitology on the Web (QP 3.0; http://www.zoologia.hu/qp/).

## RESULTS

### Epidemiological analysis of *Ophiotaenia* sp. infection

Parasitological examination revealed that *Ophiotaenia* sp. infection occurred in 5 out of 85 examined *A. kassasii* frogs, yielding an infection rate of 5.9% (95% confidence interval [CI]: 1.93%–13.20%). Although the prevalence was relatively low, the mean intensity among infected frogs was remarkably high, with an average of 70 parasites per host (95% CI: 35.00–90.00). This indicates a substantial parasite burden in the few frogs that harbored infections. The mean abundance, representing the average number of parasites across all sampled frogs (infected and uninfected), was 4.12 (95% CI: 1.18–8.82) (Table 1).

**Table 1:** Descriptive epidemiological statistics of *Ophiotaenia* sp. infection in frogs, including infection rate, mean intensity, abundance, and 95% confidence interval (CI).

Parasite	Number of infected frogs	Infection rate (95% CI)	Intensity mean (95% CI)	Mean abundance (95% CI)
*Ophiotaenia* sp*.*	5	5.9 (1.93–13.20)	70 (35.00–90.00)	4.12 (1.18–8.82)

### Morphological characteristics of recovered tapeworms

**Figure 2:**
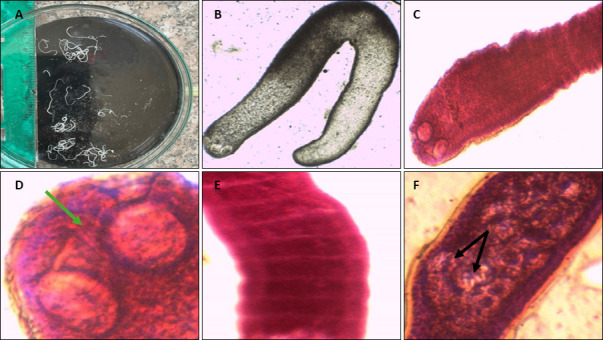
Morphological features of *Ophiotaenia* sp. outlining: (A) Gross morphology. (B) Unstained whole mount showing the scolex. (C) Magnified photomicrographs of *Ophiotaenia* sp. stained with acetic acid-alum carmine showing the scolex and two suckers (black arrows) (x40). (D) Two suckers and the apical organ (green arrow) (x100). (E) Mature segments are broader than long (x100). (F) Gravid segments are filled with eggs (x100).

The recovered adult tapeworms measured 2–30 mm in length and 0.7–0.9 mm in width. The scolex displayed two prominent spherical suckers accompanied by a distinct apical organ. Mature proglottids exhibited a length-to-width ratio of 0.555–0.525, whereas gravid proglottids were filled with approximately 25–30 eggs per segment (Figure 2). A comparative morphometric analysis showed notable similarities and differences relative to previously described *Ophiotaenia* species (Table 2) [7, 19].

**Table 2:** Measurements of *Ophiotaenia* sp. isolated from *Amietophrynus kassasii* in this study compared with previous studies.

Species	*Ophiotaenia* *saphena*	*Ophiotaenia saphena*	*Ophiotaenia* sp.	*Ophiotaenia saphena*	*Ophiotaenia* sp.
Host	*Rana clamitans*	*Rana clamitans*	*Rana pipiens*	*Rana catesbeiana*	*Amietophrynus* *kassasii*
Region	Michigan, USA	Wisconsin, USA	Wisconsin, Mississipi, USA	Arkansas, USA	New Valley Governorate, Egypt
Reference	Osler [19]	Scholz *et al*. [7]	Scholz *et al*. [7]	Scholz *et al*. [7]	Present study
Total length (mm)	40–280	108	39–42	72–119	12–30
Maximum width (mm)	1.59	0.90	0.70–0.86	0.89	0.7–0.9
Mature proglottid (length/width ratio)	1.07	N/A	N/A	0.84–1.22	0.555–0.525
Gravid proglottid (length/width ratio)	1.19	N/A	N/A	1.61–2.46	1.8–2.85
Scolex width (μm)	270–320	390–470	295–370	340–425	170–250
Scolex length (μm)	200–250	290–410	185–275	245–310	150–170
Sucker width (μm)	120–150	175–200	115–180	155–175	135–165
Apical organ	Present (degenerate)	Present	Present	Absent	Present
Apical organ width (μm)	23	95–130	75–130	Not applicable	75–105
Relative length of the cirrus sac	About 17%	22%	19%–27%	19%–23%	10%–15%
Genital pore (position)	Approximately 33% (23%–26%)	28%	17%–21%	16%–22%	20%

**Figure 3:**
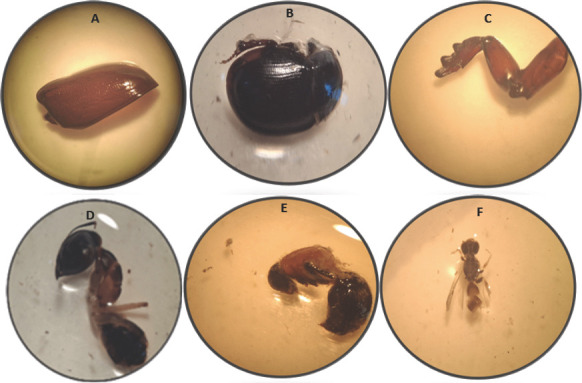
Multiple insect fragments isolated from the infected frogs showing: (A) The elytra wing of a ground beetle. (B) Body of the darkling beetles. (C) Digging leg of a mole cricket. (D) Body of an ant. (E) A bee. (F) A wasp. (x40).

Examination of the intestinal contents of infected frogs revealed multiple insect fragments, suggesting ingestion of potential intermediate or paratenic hosts. These included coleopteran parts (elytra of ground beetles and bodies of darkling beetles), orthopteran structures (legs of mole crickets), and hymenopteran remains (ants, bees, and wasps) (Figure 3).

### Chemical profiling of *Sinularia* sp. extract and the *in vitro* anticestodal activity

**Figure 4:**
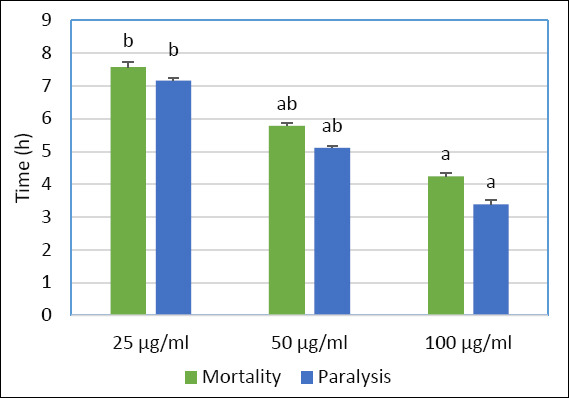
*In vitro* anticestodal effect of different concentrations of *Sinularia* sp. extract on adult stages of *Ophiotaenia* sp. in terms of mortality and paralysis.

Preliminary chemical screening of the methanolic extract of *Sinularia* sp. indicates the presence of glycosides, sterols, and diterpenes (Table-3). The methanolic extract of *Sinularia* sp. demonstrated dose-dependent anticestodal efficacy. Mortality of adult tapeworms occurred at 7.58 ± 0.15 h and 5.79 ± 0.08 h when exposed to 25 µg/mL and 50 µg/mL concentrations, respectively. The highest concentration, 100 µg/mL, induced significantly faster mortality at 4.247 ± 0.09 h, whereas tapeworms in the control group remained active for 70.39 ± 1.23 h (Table 4 and Figure 4).

**Table 3:** Preliminary chemical screening results of the methanolic extract of *Sinularia* sp.

No.	Chemical constituents	Results
1	Carbohydrates/glycosides	+
2	Flavonoids	−
3	Sterols	+
4	Diterpenes	+
5	Anthraquinones	−
6	Saponins	−
7	Tannins	−
8	Alkaloids	−
9	Cardiac glycosides	−
10	Coumarins	−

+ = present and − = absent

**Table 4:** Mortality and paralysis of adult *Ophiotaenia* sp. after incubation with different concentrations of *Sinularia* sp. extract.

Parameter	Control group (tapeworms treated with PBS)	25 µg/mL	50 µg/mL	100 µg/mL
Mortality (%)	70.39 ± 1.23ᵃ	7.58 ± 0.15ᵇ	5.79 ± 0.08ᵃᵇ	4.247 ± 0.09ᶜ
Paralysis (%)	67.77 ± 1.52ᵃ	7.16 ± 0.09ᵇ	5.12 ± 0.05ᵃᵇ	3.40 ± 0.11ᶜ

PBS = Phosphate-buffered saline. ^a–c^Means with different superscript letters are significantly different (p < 0.05). Values are presented as mean ± standard error.

### Ultrastructural alterations observed by SEM

SEM analysis of control tapeworms revealed the typical morphology of *Ophiotaenia* sp., including a scolex with four spherical suckers and a cirrus sac containing a large central cavity (~25 × 45 μm). Mature proglottids displayed clear genital pores.

In contrast, tapeworms treated with 100 µg/mL *Sinularia* sp. extract exhibited profound structural damage. Observed alterations included:

- Constriction of the cirrus sac cavity
- Shrinkage of two suckers and invagination of the remaining two
- Presence of calcareous corpuscles and vitelline cells in gravid segments
- Tegumental peeling on one side of the proglottids
- Pronounced surface erosion, wart-like projections, and segment contraction
- Genital pores positioned closer together due to proglottid distortion.

**Figure 5:**
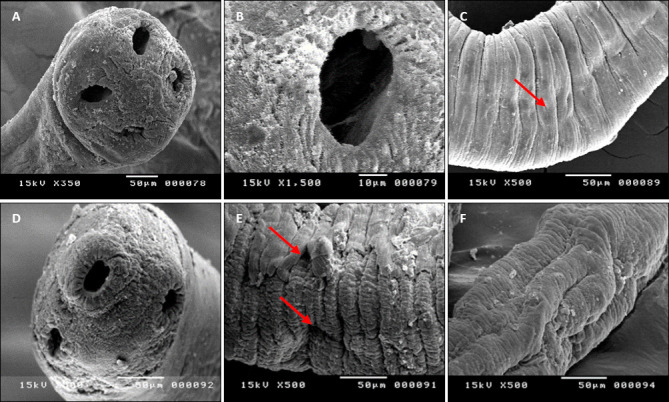
Scanning electron microscopy photomicrographs of *Ophiotaenia* sp. showing: (A–C) The control group of *Ophiotaenia* sp. (A) The scolex with four spherical suckers. (B) The cirrus-shaped sucker has a large central cavity measuring approximately 25 × 45 μm. (C) Mature segments with the genital pore (red arrow). (D-F) The treated group *Ophiotaenia* sp. (100 µg/mL). (D) The cirrus with a narrow cavity in the scolex, shrunken in two suckers, and invaginated in another two suckers. (E) Mature segments showing eroded tegmental surface, conspicuous warts, and eruptions are visible on every segment, and contraction caused the genital pores of two segments to be located at the same level due to alteration in their site (red arrows). (F) Gravid proglottids appear to be severely shrunken with acicular and capilliform filitriches.

These findings confirm that *Sinularia* sp. extract causes severe tegumental disruption, compromising essential structural and functional features of the cestode (Figure 5).

## DISCUSSION

### Epidemiological significance of *Ophiotaenia* sp. infection

This study provides the first confirmed isolation and identification of *Ophiotaenia* species infecting *A. kassasii* in the New Valley Governorate of Egypt. The infection rate observed (5.9%) was relatively low; however, the mean intensity of 70 parasites per infected frog indicates a substantial parasitic burden, suggesting that although few frogs were affected, those individuals likely experienced considerable physiological stress. This pattern reflects the characteristic epidemiology of helminths, where a minority of hosts harbor disproportionately high parasite loads, a form of aggregated distribution frequently reported in natural amphibian populations.

Reported prevalence of *Ophiotaenia* species varies widely across regions and host species, with some studies documenting extremely low infection rates (0.41%–3%) [20], while others report much higher levels, such as the 75% infection rate in *Telmatobius dankoi* from the Andes [21]. These differences are influenced by environmental conditions, host behavior, and availability of intermediate hosts. As amphibian populations decline worldwide due to habitat alteration, climate change, and anthropogenic pressures [22], understanding parasite–host dynamics becomes increasingly critical. Human encroachment into wildlife habitats and increased contact with amphibians and reptiles also raise concern about potential zoonotic risks, although such risks remain largely hypothetical for *Ophiotaenia* species [23].

### Morphological characteristics and host–parasite associations

Members of the family Proteocephalidae, including the genus *Ophiotaenia* La Rue, 1911, typically parasitize reptiles and amphibians. Although generally host-specific, *Ophiotaenia* species have been documented in various congeneric hosts across multiple continents [4, 5, 8, 24–26]. In the present study, adult tapeworms measured 12–30 mm × 0.7–0.9 mm, possessing a scolex with two spherical suckers and a distinct apical organ. Mature proglottids displayed a length-to-width ratio of 0.555–0.525, and gravid segments contained numerous eggs. Morphometric comparisons revealed several differences from previously described species by Scholz *et al*. [7] and Osler [19].

Analysis of intestinal contents from infected frogs revealed fragments of coleopteran, orthopteran, and hymenopteran insects, suggesting their potential role as intermediate or paratenic hosts. Despite the importance of these hosts, the life cycles of proteocephalidean tapeworms remain poorly understood. Most *Ophiotaenia* species are believed to require two intermediate hosts, starting with small aquatic or terrestrial insects that harbor larval stages. Some species, such as those infecting *Ameiurus nebulosus*, may utilize paratenic hosts [8]. Although no human infections have been reported, maintaining proper hygiene when handling wild animals or insects is recommended [25].

Transmission pathways for species infecting terrestrial vertebrates, such as *Thaumasioscolex didelphidis*, remain largely unknown, though frogs, fish, and insects are believed to mediate transmission [27]. In temperate aquatic systems, many proteocephalids rely solely on copepods as intermediate hosts, wherein the plerocercoid stage develops [8, 28].

### Diversity and phylogenetic considerations

Proteocephalidean cestodes in Africa most commonly infect snakes and catfish. In Egypt, *Ophiotaenia tessellata* has been recorded in *Natrix tessellata* [29]. Phylogenetic analyses indicate that *Ophiotaenia* is polyphyletic, comprising morphologically similar yet distantly related taxa, likely due to convergent evolution [29]. Overall, the diversity, host range, and life cycles of proteocephalids in anurans remain underexplored. Research limitations stem from declining amphibian populations, protection status, and the lack of comprehensive morpho-molecular characterizations. Moreover, the absence of experimental infections and histological analyses restricts the understanding of parasite impacts on native and non-native hosts [26].

### Anticestodal activity of *Sinularia* sp. extract

The methanolic extract of *Sinularia* sp. exhibited clear dose-dependent anticestodal activity against adult *Ophiotaenia* species. Incubation with increasing concentrations of the extract significantly reduced parasite motility, with the fastest mortality observed at 100 µg/mL (4.247 ± 0.09 h). SEM examination demonstrated severe ultrastructural damage in treated specimens, including narrowing of the scolex cirrus, pronounced tegumental erosion, and architectural disruption of proglottids. In contrast, control tapeworms retained intact scoleces, suckers, and tegumental surfaces.

The tegument plays a vital role in nutrient absorption, immune evasion, osmoregulation, and protection from host digestive enzymes. Thus, structural disruption directly interferes with parasite survival. Effective anthelmintics typically target the tegument [30, 31], supporting the strong cestocidal potential observed in *Sinularia* sp. extract.

### Bioactive potential of marine-derived compounds

In the current study, preliminary chemical screening of the methanolic extract of *Sinularia* sp. indicates the presence of glycosides, sterols, and diterpenes*. Sinularia*, a widely distributed genus of soft corals, consists of interconnected polyps forming a colonial organism. They produce diverse bioactive metabolites, including sesquiterpenes, diterpenes, norditerpenes, polyhydroxylated steroids, and polyamines, with demonstrated antimicrobial, antifungal, and anti-inflammatory effects [32, 33]. Essential oils from *Sinularia* species have also been isolated from samples collected in the Iranian coastline [34]. Numerous studies [35–38] support the anthelmintic efficacy of marine-derived phytochemicals, often in a dose-dependent manner. For instance, phenolic compounds from *Acanthophora* spp. inhibit *Haemonchus contortus* larvae [39], while *Bifurcaria bifurcata* extracts show activity across developmental stages of *Heligmosomoides polygyrus bakeri* [40]. A halogenated β-bisabolene sesquiterpenoid from *Laurencia scoparia* demonstrated potent antinematodal activity against L4-stage *Nippostrongylus brasiliensis* [41], emphasizing the therapeutic promise of marine natural products.

## CONCLUSION

This study provides the first confirmed report of *Ophiotaenia* sp. infection in *A. kassasii* frogs in the New Valley Governorate of Egypt, establishing essential baseline data for amphibian parasitology in the region. Although the overall prevalence was low (5.9%), infected frogs carried a remarkably high mean intensity of 70 parasites per host, indicating a substantial trematode burden capable of impairing host physiology and potentially affecting population health. Detailed morphological and SEM examinations confirmed key diagnostic features of the species, while gut-content analysis, revealing coleopteran, orthopteran, and hymenopteran fragments, supports the role of insect taxa in parasite transmission.

The methanolic extract of *Sinularia* sp. demonstrated strong dose-dependent anticestodal activity, with the highest concentration (100 µg/mL) producing the fastest mortality of adult tapeworms (4.247 ± 0.09 h). SEM findings revealed extensive tegumental damage in treated worms, including sucker shrinkage, cirrus cavity constriction, and severe erosion of proglottids, highlighting the extract’s potent cestocidal properties. These observations underscore the promising therapeutic potential of marine-derived natural compounds as alternative anthelmintic agents.

The study’s strengths include being the first regional documentation of *Ophiotaenia* in *A. kassasii*, the combined use of light microscopy and SEM for accurate morphological validation, the incorporation of ecological evidence through gut-content analysis, and the clear experimental demonstration of the cestocidal effects of *Sinularia* sp. extract. However, certain limitations remain, including the geographically restricted sample, the absence of molecular identification for species-level confirmation, the lack of seasonal prevalence data, and the reliance on *in vitro* assays, which may not capture the full complexity of host–parasite interactions.

Future research should incorporate molecular sequencing to resolve phylogenetic placement, broaden sampling across multiple habitats and seasons, and investigate the full life cycle to identify intermediate and paratenic hosts. *In vivo* trials are also needed to validate the safety and efficacy of *Sinularia* extracts, along with chemical characterization studies to isolate and identify the specific bioactive compounds responsible for cestocidal activity.

Overall, this study advances the understanding of cestode infections in Egyptian amphibians and highlights the potential of *Sinularia* species as a promising source of natural anthelmintic agents. These findings contribute valuable insights into regional biodiversity, parasite ecology, and the future development of marine-derived antiparasitic therapies.

## DATA AVAILABILITY

All the generated data are included in the manuscript.

## AUTHORS’ CONTRIBUTIONS

BSA, MA, AA, MAT, and FASA: Conceptualized the study, methodology, and manuscript preparation. HA, GDZ, and GEB: Formal analysis and drafted the manuscript. All authors have reviewed and approved the final version of the manuscript.
